# The establishment of Health and Demographic Surveillance system (HDSS) in Bauchi State North East Nigeria; baseline findings, challenges and lessons learned

**DOI:** 10.1080/16549716.2026.2710938

**Published:** 2026-09-01

**Authors:** Idris Muhammad Bose, Muhammad Mubarak Dabo, Muhammad Abdullahi Kobi, Abubakar Musa, Saad Ibrahim Amaya, Awwal Dahiru, Jamilu Yaya, Lamaran Makama Dattijo, Jonathan A. Muir, Muhammad Faruk Bashir

**Affiliations:** a Child Health and Mortality Prevention Surveillance CHAMPS, Health and Demographic Surveillance System Unit, Abubakar Tafawa Balewa University, Bauchi, Nigeria; b Child Health and Mortality Prevention Surveillance CHAMPS, Information and Communication Technology Unit, Abubakar Tafawa Balewa University, Bauchi, Nigeria; c Department of Community Medicine, Abubakar Tafawa Balewa University, Bauchi, Nigeria; d Child Health and Mortality Prevention Surveillance CHAMPS, Community Engagement Unit, Abubakar Tafawa Balewa University, Bauchi, Nigeria; e Department of Obstetrics and Genecology, Abubakar Tafawa Balewa University, Bauchi, Nigeria; f CHAMPS Program Office, Task Force for Global Health, Emory University, Atlanta, USA; g Department of Pediatrics, Abubakar Tafawa Balewa University, Bauchi, Nigeria

**Keywords:** Baseline enumeration, population composition, population health, Bauchi, Nigeria

## Abstract

**Background:**

Bauchi State has a weak civil and vital registration system; hence the need for robust surveillance system. The Bauchi Health and Demographic Surveillance System (HDSS) was established in 2023 with support from Child Health and Mortality Prevention Surveillance (CHAMPS) network. Its purpose is to obtain reliable population data.

**Objectives:**

To establish a baseline demographic and health profile of the target community and to illustrate the feasibility and challenges of developing a high-quality HDSS in a resource-limited setting in Nigeria characterized by high mortality rates.

**Methods:**

An initial household enumeration was conducted in 257 enumeration areas across eight contiguous administrative wards within the Bauchi and Ganjuwa Local Government Areas of Bauchi State. Following community awareness and field re-demarcation, prospective longitudinal follow-up was commenced.

**Results:**

A total of 151,672 individuals in 23,476 households were enrolled. The population structure reflects a young population with 53.3% <15 years. The total dependency ratio is 104. Socioeconomic indicators show low formal education attainment and high unemployment (49%). Household conditions reveal substantial reliance on solid cooking fuels (78%) and on-site pit latrines. Implementation challenges included outdated maps, initial community mistrust, and data system instability.

**Conclusion:**

The Bauchi HDSS successfully established a surveillance framework which identified a young population with significant public health concern. It shows that it is possible to establish demographic surveillance system in a place with high mortality by using targeted strategies like getting the community involved, hiring local staff, re-demarcating the field, and building technical capacity.

## Paper Context

**Main findings:** The study established a health and demographic surveillance system in Bauchi State, enrolling 151,672 individuals across23,476households as a baseline population, initiating scheduled follow-up rounds, and identifying a young population with substantial socioeconomic and environmental health risks.
**Added knowledge:** The paper demonstrates that longitudinal population surveillance can be implemented in a resource-limited, high-mortality setting and the study provide a platform for pregnancy surveillance, mortality surveillance and verbal and social autopsy including platform for other future nested studies.
**Global health impact for policy and action:** The findings provide locally generated evidence to inform maternal and child health policies, and support the use of surveillance systems for data-driven public health interventions in similar settings.

## Background

The global burden of disease and premature mortality remains a significant challenge for public health, particularly in low- and middle-income countries (LMICs). To identify and understand the determinants influencing population health outcomes and their temporal variations, it is essential to have reliable and continuous data on fertility, morbidity, mortality, migration, and other socio-demographic characteristics for distinct demographic groups. However, in most sub-Saharan African countries, including Nigeria, the absence of functional and comprehensive vital registration systems has severely hindered the collecting of essential data. Efforts to address this gap have facilitated the establishment of demographic surveillance systems that continuously monitor demographic events and provide precise estimates of population changes in resource-limited areas.

Demographic Surveillance Systems (DSSs) are enduring population-centric frameworks that collect, refresh, and evaluate data regarding births, deaths, migrations, and socio-economic characteristics within geographically delineated Demographic Surveillance Areas (DSAs [1] A HDSS is a platform that integrates health event monitoring, either passively via facility-based data collection or actively through community-based systems, with demography tracking [1]. HDSS sites originated in mid-20th-century population research aimed at remedying the deficiency of reliable demographic data in emerging nations. Over time, these systems have developed into robust networks that deliver health policy, implement direct interventions, and assess results using population-based data consistently [2]. The International Network for the Demographic Evaluation of Populations and Their Health (INDEPTH Network) was established in 1998 to standardize methodologies, promote collaboration, and ensure comparability of data from HDSS sites globally [2] Presently, more than 45 HDSS sites operate in sub-Saharan Africa and South Asia, tracking over three million persons. This information aids in generating valuable long-term statistics for global health and development planning [3].

Nigeria is a lower-middle-income nation in West Africa, with an approximate population of 238 million and a population density of 261 individuals per square kilometer [4]. The civil registration and vital statistics (CRVS) system in Nigeria, the primary mechanism for documenting births, deaths, and marriages, is insufficient, disjointed, and underutilized, notwithstanding the nation’s substantial population and health requirements. Evidence suggests that over 70% of the nation’s five million annual births are unregistered, and reliable national records for death and marriage registration are absent [5]. Consequently, CRVS data are frequently incomplete, delayed, and inaccurate, rendering them less effective for policy formulation, health monitoring, and program evaluation, eventually obstructing efficient public health interventions and resource distribution in Nigeria.

HDSS have been established in several regions of Nigeria to obtain precise, community-based demographic and health data, addressing significant data deficiencies. Prior to the establishment of the Bauchi HDSS, two other HDSS sites were operational: the NAHUCHE HDSS and the Cross River HDSS. The NAHUCHE HDSS was established in 2009 in the Bungudu Local Government Area of Zamfara State, located in north-west Nigeria. It has more than 100 communities in six districts, with a population of 137,823 inhabitants. The population structure is predominantly youth [6,7]. The Cross River HDSS was established in 2010 in the Akpabuyo Local Government Area of Cross River State, located in southern Nigeria. The site operates two research cohorts located within the southern senatorial district of Cross River State in south-south Nigeria, with a combined population of 37,808 persons in 9,452 households as at 2018 (about 47.4% of which are rural dwellers). Its primary aim was to address the near-total absence of reliable systems for collecting community-based data to feed into Nigeria’s national health information and vital registration systems [8]. These HDSS sites were specifically designed to fill the data void left by weak Civil Registration and Vital Statistics (CRVS) structures, providing longitudinal data to support research, policy development, and public health planning.

Despite improvements in the health system, the north-east region of Nigeria remains one of the most underserved areas in terms of documenting critical events and health data, exacerbating the persistent challenges of elevated mortality rates and suboptimal health outcomes. The 2022 Multiple Indicator Cluster Survey (MICS) and the 2023 Nigeria Demographic and Health Survey (NDHS) indicate that the North-East region exhibits some of the highest death rates in sub-Saharan Africa. The under-five mortality rate is 127 deaths per 1,000 live births, exceeding the national average of 102 deaths per 1,000 live births. The mortality rates for infants and neonates are 66 and 40 per 1,000 live births, respectively [9,10]. Bauchi State, specifically, continues to face numerous health challenges. The HIV prevalence is 2.3%, indicating that about 53,000 individuals are infected. The incidence of non-communicable diseases is escalating, particularly among urban populations, with diabetes, hypertension, and cardiovascular disorders becoming ever more common. The maternal mortality rate remains significantly elevated, at 1,549 deaths per 100,000 live births. The mortality rates for children under five, infants, and newborns are 147, 69, and 38 deaths per 1,000 live births, respectively. Women in Bauchi typically have an average of eight children with an average interval of 30 months between births. Only 21% of deliveries occur in health facilities, about one-third (33%) of women receive no antenatal care. Furthermore, only 11% of children aged between 2–23 months have received vaccinations [10]. These statistics underscore the urgent need for a reliable, locally adaptive surveillance mechanism to guide health planning and evaluate interventions.

The establishment of the Bauchi-HDSS is a strategic approach to address the significant data deficiency in north-east Nigeria. The Bauchi HDSS is a comprehensive system for gathering data on demographic changes, fertility, mortality, migration, and household attributes across geographically defined communities in Bauchi and Ganjuwa Local Government Areas. The HDSS employs real-time monitoring, community engagement, and rigorous data quality assessments to ensure data completeness and accuracy, in contrast to the disjointed national CRVS. This article presents key demographic data from the Bauchi HDSS baseline enumeration as well as a discussion of the major problems encountered, and lessons learned throughout the implementation of the Bauchi HDSS baseline enumeration operation.

### Significance of the study

The establishment of the Bauchi-HDSS is crucial for public health research at both local and global levels. At the local level, it provides a dependable and enduring method for gathering long-term evidence to assist in the planning, implementation, and evaluation of health and social programs in Bauchi State and throughout North-East Nigeria. The HDSS serves as an effective method for obtaining samples for epidemiological investigations and provides essential data for calculating health and death rates. This enhances the accuracy of population-based estimates. It enhances Nigeria’s national health information system by addressing deficiencies in the inadequate CRVS framework and facilitating evidence-based policy formulation. Globally, the Bauchi HDSS adds to the growing network of INDEPTH-affiliated sites generating comparable data for tracking progress towards the SDGs target, particularly those related to maternal and child health.

This paper documents the procedures undertaken to establish the Bauchi HDSS, presents significant baseline findings, discusses challenges encountered during its implementation, and extracts critical lessons that may inform the design and sustainability of future demographic and health surveillance systems.

## Methodology

### Aims of Bauchi HDSS

The Bauchi-HDSS was founded in October, 2023 in an effort to establish the Child Health and Mortality Prevention Surveillance (CHAMPS) Project in Nigeria. CHAMPS is a multi-country program working in nine countries in sub-Saharan Africa and some part of South Asia [2]. The key objective of CHAMPS is to define population-based rates for definitive causes of death through gold standard diagnostic and laboratory methods nested within population surveillance. The HDSS sites in CHAMPS provide this platform by generating household and demographic data for estimating population-based mortality rates and related information in order to identify causes of deaths among children < 5 years of age [1].

The Bauchi-HDSS conducts a longitudinal follow up of household members using two data collection rounds of six months duration each to collect information on households and individual characteristics, socio-economic status, mother characteristics including pregnancy history and pregnancy outcomes, newborn characteristics including immunization uptake and anthropometric measurement as well as migration and death records.

### The study area

The Bauchi HDSS is located in Bauchi State North-east Nigeria, with the central office located at the college of Medical Sciences, Abubakar Tafawa Balewa University, Bauchi. Bauchi State is located between latitudes 9°3 and 12°3 North and longitudes 8°50′ and 11° East. Based on 1991 census, the state had a population of 3,836,333 people, which has since grown substantially in subsequent estimates to 8,308,800 Population in 2022 [11]. The state spans two main vegetation zones the Sudan and Sahel savannahs. The southern part, influenced by the Jos Plateau, is mountainous and greener with an annual rainfall of about 1300 mm, while the northern region is drier, sandy, and semi-desert-like, receiving around 700 mm. Weather conditions vary considerably, with humid heat in the south and hot, dry, dusty conditions in the north. Bauchi is culturally diverse, hosting 55 ethnic groups such as the Hausa, Fulani, Gerawa, and Sayawa, who share similarities in language, occupation, and cultural practices. Administratively, the state is divided into three senatorial zones, twenty local government areas, and six emirates. Situated in Bauchi and Ganjuwa Local Government areas [12].

The HDSS site in Bauchi is divided into an urban and a rural catchment. The urban catchment area is located within the state capital across three contiguous political Wards in Bauchi Local government namely; Kundum Durum, Miri and Birshi spanning across 15 communities and semi urban villages. While the rural catchment area is located at Ganjuwa LGA which is 30 Km away from the state capital. The Ganjuwa catchment area includes 8 contiguous administrative wards of Kariya A, Miya A, Miya C, Kafin Madaki A and Kafin Madaki B spanning in 2 major towns and over 38 villages. The HDSS Site is sub-divided into 18 operational clusters across the urban and rural catchment area. 9 non-contiguous clusters in Bauchi and 9 Contiguous clusters in Ganjuwa LGA formed part of the study area.

**Figure 1.**
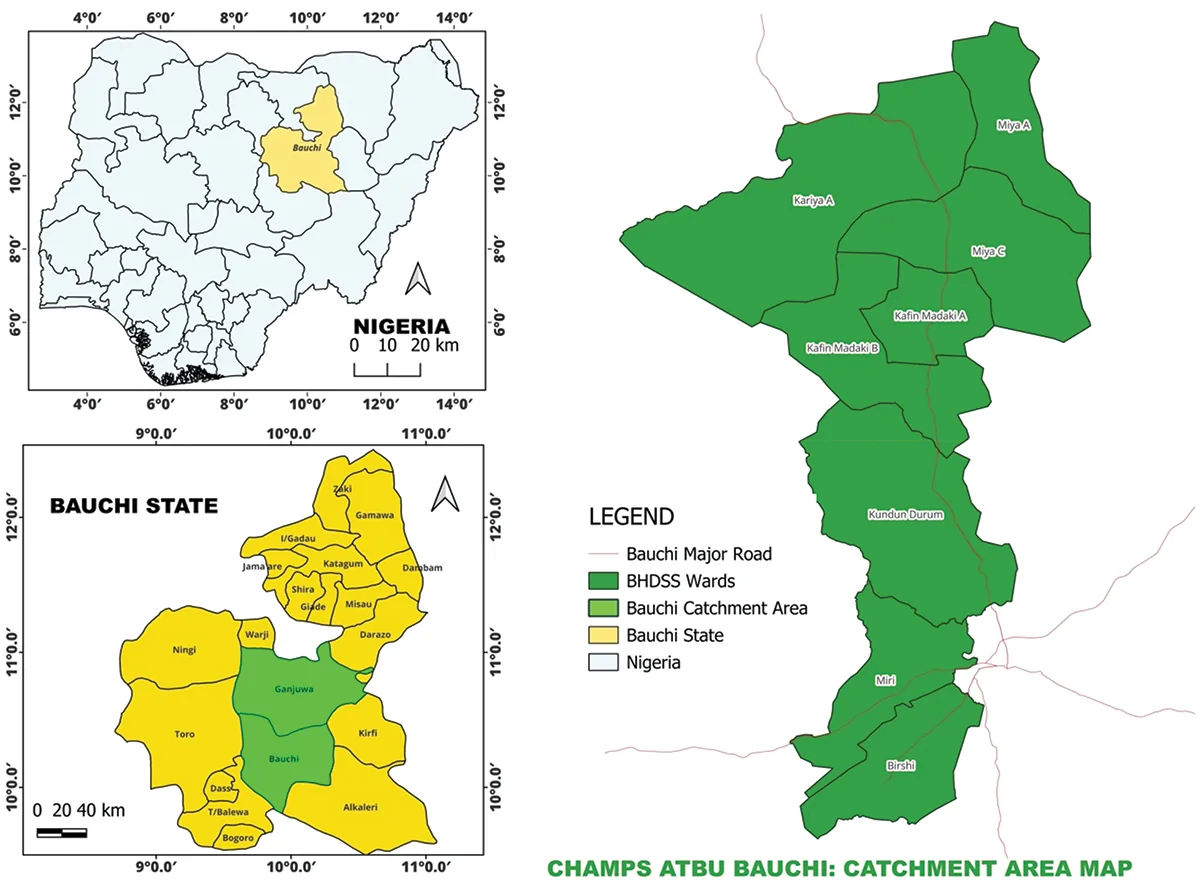
Bauchi HDSS study area.

Figure 1 shows the location of the Health and Demographic Surveillance System across the two Local Government Areas and the eight selected wards. It provides a clear visual of the surveillance coverage at the ward level, illustrating the administrative areas included in the baseline enumeration and follow-up activities.

### Ethical consideration

Ethical approval for the establishment and operation of the Bauchi-HDSS was obtained from the Health Research Ethics Committee (HREC) of Bauchi State Ministry of Health (SMoH). The study adheres strictly to national and international ethical standards for research involving human participants. The study was carried out in accordance with the principles of the Helsinki declaration. Written informed consent was obtained from each participating household prior to data collection. All respondents were adults 18 years and above, and participation was entirely voluntary. Participants were adequately informed about the study objectives, procedures, potential risks, and benefits before consent was obtained. Confidentiality and privacy of respondents were strictly maintained throughout the study.

### Recruitment and training

Following training conducted by the CHAMPS Program Office HDSS Lead in September, 2023 at the University of Calabar, recruitment for the Bauchi HDSS supervisors and enumerators commenced through an open advertisement requiring a minimum of a diploma qualification, with preference given to candidates with relevant experience or health-related backgrounds. Approximately 100 applicants mainly residents of the catchment area were shortlisted for interviews, leading to the selection of 75 data collectors, 63 enumerators, and 12 supervisors. In October 2023, a comprehensive training was held, focusing on mapping and listing procedures, theoretical sessions on the baseline standardized questionnaire, and a field pilot exercise. Pre- and post-tests were conducted to evaluate performance, resulting in the final selection of 70 participants (10 supervisors and 60 enumerators) for field deployment. The training and pilot sessions were jointly facilitated by the HDSS lead and Co-lead, Field Research Officers (GIS and Operations), the ICT Lead and Co-Lead, and the Data Manager.

### Baseline census

The Bauchi-HDSS baseline census was launched after preparatory activities, including staff training and mapping with the support of National Population Commission (NPC) Staff. Household mapping, listing and baseline enumeration were conducted across 213 enumeration areas obtained from the NPC in Bauchi and Ganjuwa LGAs between December 2023 and May 2024. Following this, a supplementary baseline enumeration covering an additional 44 enumeration areas was carried out from May to July 2025 to include previously uncovered locations. The exercise engaged 18 data collectors and six supervisors after a refresher training session. The entire process was coordinated by the HDSS Lead and supervised by the Field Research Officers, Data Manager, and ICT Lead/Co-Lead to ensure data quality, completeness, and adherence to standard protocols.

### Data quality

To ensure data accuracy, the Data Manager implemented SQL-based data quality queries that automatically flagged errors in uploaded records. Supervisors conducted back-checks on selected households to verify data consistency. The FROs performed spot checks on both data collectors and supervisors to assess field performance. This multi-level system ensured continuous validation and high-quality data throughout the HDSS enumeration process.

Table 1 outlines a three-level structure of data elements collected within the surveillance system. Level 1 captures core demographic indicators essential for monitoring population size, fertility, and mortality, while Level 2 provides household, maternal, and child characteristics that contextualize health and mortality patterns. Level 3 includes specialized modules on anthropometric measurements and contraceptive uptake to support more targeted public health analysis and interventions.

**Table 1.** Data element in Bauchi health and demographic surveillance system.

Level of Data Element	Description
Level 1	Key priority variables that are fundamental to meeting CHAMPS objectives. These include age- and sex-specific population size, fertility and mortality by age and sex.
Level 2	Household and individual characteristics important for contextualizing CHAMPS findings including: (1) Household socioeconomic status; access to water, electricity and sanitation; cooking fuel; access to healthcare; and living arrangement. (2) Mother characteristics - age, education, marital status, religion, and reproductive history. (3) Child characteristics: antenatal care, delivery, postnatal care, immunization uptake.
Level 3	Anthropometric measurement and Contraceptives uptake.

### Analysis and statistical methods

We adopted a descriptive analysis using baseline data from the Bauchi-HDSS. The dependent variables analyzed included proportion of under-five children to the total population, Dependency ratio, proportion of the aged population (≥65 years), sex ratio at birth, and average household size. These variables were computed using standard demographic formulas. Descriptive statistics were applied to analyze independent variables such as age, sex, education, employment, marital status, source of drinking water, toilet facility and cooking fuel. The age-sex distribution of the population was presented using a population pyramid, while other variables were summarized in tables and percentage distributions. Data were entered into an excel spread sheet for cleaning and coding then analyzed using STATA version 17. The results were presented in tables and figures to describe the baseline findings and health characteristics of the Bauchi HDSS population.

## Results

This analysis delineates the fundamental socio-demographic, household, and Water, Sanitation, and Hygiene (WASH) profile of the Bauchi HDSS cohort. The surveillance platform, as outlined in Table 2, encompasses a dynamic population of 151,672 individuals living in 23,476 dwellings, distributed over an area of about 591 km^2^ within its operational clusters. Significant demographic sub-groups of public health relevance comprise 39,175 women of reproductive age and 25,059 children under five years old.

**Table 2.** Population under surveillance in Bauchi-HDSS catchment area.

S/N	Cluster	Number of Households	Total Population	Women of Reproductive Age	Total Live Births	Total Under 5 Children
1	Kafin Liman	1,272	7,924	1,798	153	1,679
2	Durum	1,115	7,382	1,750	254	1,340
3	Birshi Gandu	1,716	8,682	2,482	110	1,020
4	Miya SE	993	6,737	1,758	100	1,118
5	Miya NW	1,258	9,079	2,225	140	1,620
6	Bayara	1,266	7,768	2,116	26	1,196
7	Kafin Madaki East	1,575	12,731	3,400	89	1,772
8	Siri Gameru	1,560	9,563	2,237	225	1,984
9	Miri	1,133	8,170	2,084	165	1,325
10	Miya NE	922	6,335	1,648	100	1,124
11	Yolan Bayara	985	6,378	1,674	46	1,103
12	Lushi	1,042	7,177	1,894	116	1,261
13	Rafin Zurfi	1,459	5,959	1,881	42	598
14	Yelwa	879	5,689	1,588	74	753
15	Kafin Madaki West	1,604	9,925	2,374	141	1,851
16	Miya SW	1,161	8,702	2,202	119	1,387
17	Birshin Fulani	1,806	12,294	3,284	127	1,876
18	Kariya	1,730	11,177	2,780	141	2,052
	**Totals**	**23,476**	**151,672**	**39,175**	**2,168**	**25,059**

**Figure 2.**
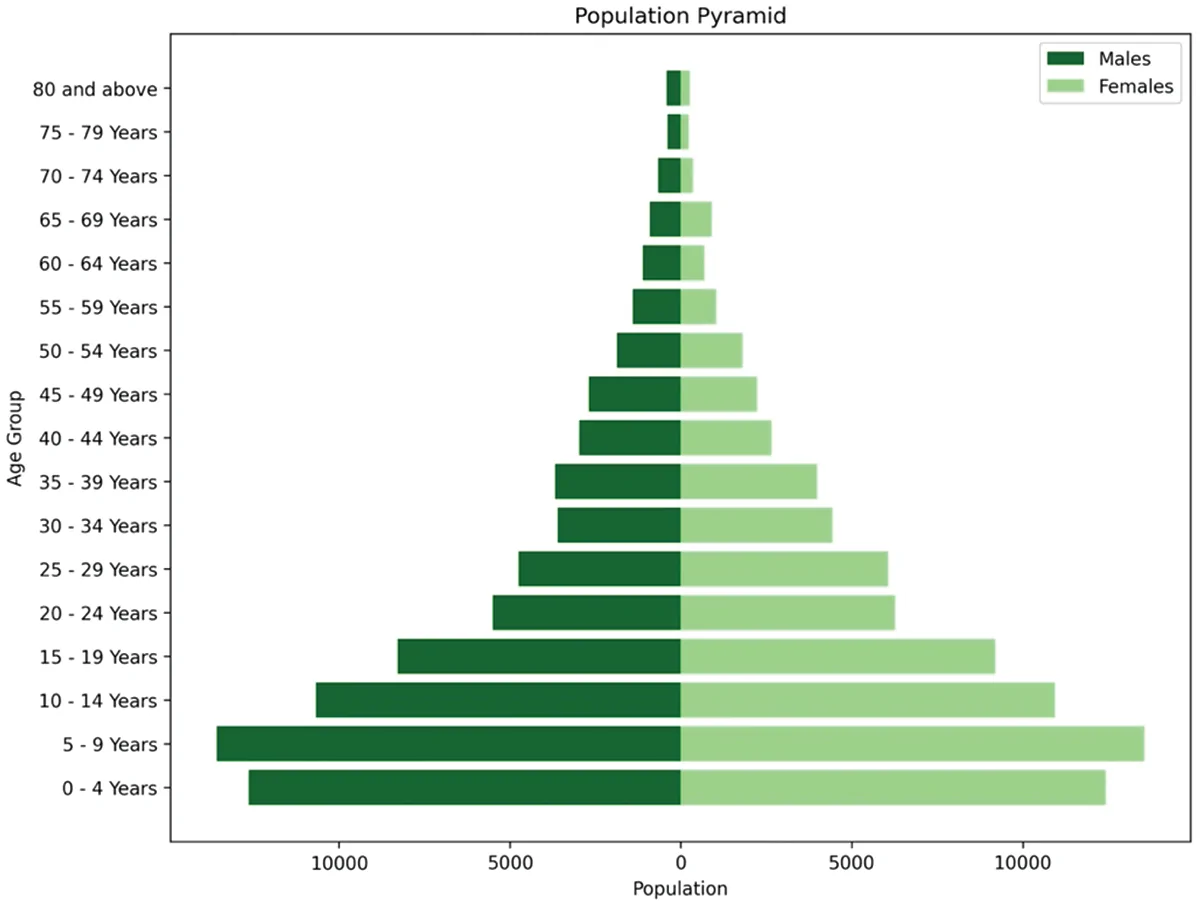
Bauchi HDSS population pyramid.

The demographic composition of the monitored population, depicted in the population pyramid with wide base (Figure 2), is indicative of a young population with 53.3% <15 years, of this −9 years constitutes 18.5%. Table 5 further quantifies that children under-five comprises 16.5% of the entire population, whilst the elderly population (65+) constitutes merely 2.4%. The sex ratio at birth is 1. The mean household size is seven individuals and the total dependency ratio of stands at 104.

**Table 5.** Key demographic Indicators from the Bauchi HDSS baseline data.

Indicators	Total (*n* = 151,672)
Proportion of under 5 children to the total population	16.5
Proportion of aged population (>65 years) to the total population	2.4
Sex Ratio at birth	100
Total Dependency ratio	104
Average Household size	7

Table 3 displays essential socio-demographic data, indicating a population with minimal formal education. Among individuals aged 12 and older, the majority lack formal education. Concerning marital and employment status, the majority of residents are married at 56%, whereas 40% are single. Unemployment stands at 48.97%, with merely 7.12% of the population classified as fully employed and 40.63% as self-employed.

**Table 3.** Distribution of population under surveillance by socio-demographic characteristics in the Bauchi-HDSS.

Variable	% of Bauchi HDSS Population (*n* = 151,672)
Sex:	
Male	49.51%
Female	50.49%
Level of education (Population aged 12+):	
No education	26.22%
Primary education	18.73%
Secondary education	24.12%
Integrated education	17.00%
Pre-ND Certificate	0.32%
ND/NCE/Nursing	8.04%
HND	1.45%
BSc/Btech/MBBS/BL	3.36%
Postgraduate, PGD/MSc/Mtech/Mphil /PhD	0.76%
Employment:	
Unemployed	48.97%
Full-time employment	7.12%
Part-time employment	3.28%
Self Employed	40.63%
Marital Status	
Single	40.47%
Married	56.00%
Cohabiting	0.00%
Divorced	0.66%
Separated	0.34%
Widow/Widower	2.47%
Others	0.04%

Table 4 encapsulates the WASH and household living condition. The predominant source of drinking water for most individuals is a tube well or borehole (31.13%), succeeded by protected wells (26.8%) and unprotected wells (21.07%). In terms of sanitation, pit latrines (either with a slab or ventilated) are the predominant facilities, with a substantial majority (64.9%) situated within the household’s premises. Household energy use is primarily reliant on solid fuels: 78% of the population utilizes wood, grass, straw, or bushes, while 13.4% employs charcoal. Cooking sites differ, with 43.7% utilizing a distinct room, 29.9% preparing meals within the main home without a separate room, and 15.2% cooking outside the main house.

**Table 4.** Distribution of Water sanitation and hygiene (WASH) Indicators.

Variables	% of WASH Indicators
**Sources of Drinking Water:**	
Pipe Into Dwelling	2.87%
Pipe Into Compound	1.65%
Pipe to Neighbor	3.24%
Pipe to Public Tap	2.39%
Tube well/Borehole	31.13%
Dug Protected Well.	26.83%
Dug Unprotected Well.	21.07%
Spring Water Protected	0.00%
Spring Water Unprotected	0.00%
Rainwater Collection	0.02%
Delivered Water (tanker/truck)	0.30%
Delivered Water (cart)	3.73%
Surface Water	2.01%
Packaged Water (bottle/leather)	0.06%
Sachet Water	4.64%
Water Kiosk	0.05%
**Cooking Fuel:**	
Alcohol/Ethanol	0.03%
Gasoline/Diesel	0.00%
Kerosine/Paraffin	0.32%
Natural Gas	6.67%
Coal/Lignite.	0.01%
Charcoal	13.40%
Wood/Grass/Straw/Shrubs.	78.54%
Agricultural Crop Residue	0.21%
Animal Waste/Dung.	0.04%
Processed Biomass	0.00%
Wood Chips, Garbage/Plastic, Sawdust	0.08%
Electricity	0.70%
**Cooking Location:**	
In Main House (No Separate Room)	29.95%
In Main House (Separate Room)	43.77%
Outside Main House (Separate Room)	8.66%
Outside Main House (Open Air)	15.25%
Outside Main House (Veranda/Covered Porch)	2.37%
**Toilet Facility:**	
Flush To Septic Tank	8.50%
Flush To Pit Latrine	10.27%
Flush To Open Drain	0.26%
Flush Don’t Know Where	0.11%
Pit Latrines Ventilated	11.59%
Pit Latrines with Slab	55.10%
Pit Latrines without Slab	10.94%
Composting Toilet	0.29%
Bucket	0.01%
Hanging Toilet	0.58%
No Facility	2.36%
**Toilet Location:**	
In Own Dwelling	64.94%
In Own Yard	32.18%
Elsewhere	2.89%
**Toilet Sharing:**	
Shared Toilet with others	10.55%
Not Shared Toilet with others	89.45%

Note: Total Population Under Surveillance (n = 23,476).

## Discussion

The Bauchi HDSS baseline profile reveals a young population, with a notable dependency ratio. In terms of population structure, the Bauchi HDSS is consistent with that of the NAHUCHE HDSS in Zamfara State Northwest Nigeria and the Cross River HDSS in southern Nigeria. Similar to Bauchi HDSS, the NAHUCHE HDSS population is characterized by a broad-based population pyramid, reflecting a majority of young population [7]. However, the Nahuche HDSS has a relatively larger proportion of children under five years (20.4%) compared with the Bauchi HDSS (16.5%). This suggests that fertility levels may be relatively higher in Nahuche or that Bauchi HDSS is undergoing a gradual demographic transition with a slightly lower proportion of young children. The proportion of older persons (≥65 years) was low in both surveillance sites, accounting for (3.0%) of the population in NAHUCHE HDSS and 2.4% in Bauchi HDSS [7]. These findings indicate that both populations are at an early stage of demographic transition and are characterized by low population ageing. The slightly higher proportion of elderly persons in NAHUCHE may reflect differences in survival patterns, migration dynamics, or population composition.

This demographic pattern reflects the reality in most of the states in northern Nigeria, including Bauchi state. The National Population Commission 2022 population projection estimate for Bauchi state represent similar pattern of population structure with children between aged 0–4 years recording (18.69%) slightly higher than the BHDSS (16.5%) [13]. Similarly, the proportion of aged population (65 years and above) is slightly lower in the BHDSS (2.4%) than in the NPC population estimate for Bauchi State (2.8%). Despite the differences, both populations exhibit a very small elderly population, reflecting a youthful age structure and low population ageing. The similarity between Bauchi and NAHUCHE HDSS indicates that both the HDSS sites are experiencing ongoing population growth and a substantial dependency burden resulting from a large youthful population. This may also reflect the similarities in terms of social and cultural characteristics of the populations.

Comparable findings have been reported from the Cross River HDSS, which also established a predominantly youthful population structure. Despite the dissimilarities in terms of geographical location and socio-cultural set-up, children aged (0–14) years accounted for approximately (35%) and (43%) of the population in the Cross River HDSS in urban and rural cohorts, respectively [14]. However, the proportion of children aged (0–14) years in the BHDSS was substantially higher, (53.3%), indicating a younger age structure, signifying higher fertility levels and sustained population growth within the surveillance area. Similarly, the dependent population (0–14 years and ≥65 years) in the Cross River HDSS ranged from (36.9%) in rural areas to (48.4%) in urban areas [14], whereas dependents constituted approximately (55.7%) of the BHDSS population. This higher dependency burden reflects the large concentration of children and adolescents within the BHDSS and has important implications for household welfare, educational demand, and maternal and child health services. Nevertheless, both HDSS sites share the common demographic characteristic of youthful populations with relatively small elderly populations, underscoring the persistence of high fertility and rapid population growth as key features of many demographic surveillance populations in Nigeria.

The Bauchi HDSS also reflects the experiences from HDSS sites in Nigeria and other low- and middle-income countries (LMICs), while also stressing several distinctive operational and demographic features. Similar to the NAHUCHE HDSS in northwest Nigeria and the South African HDSS, community engagement emerged as a critical prerequisite for successful surveillance implementation. In all the three settings, initial community mistrust and limited understanding of HDSS objectives affected the participation and acceptability of the project. However, a distinctive contribution of the Bauchi HDSS relates to the operational challenges encountered during establishment. Unlike NAHUCHE and the South African HDSS, where major concerns centered on fatigue, in-security, staff retention, and inaccessible households, the most significant challenge in Bauchi was the reliance on outdated 2005 Enumeration Area (EA) maps. This resulted in omitted settlements, overlapping boundaries, and potential duplication of households during baseline enumeration. The challenge underscores a broader limitation affecting demographic surveillance and census-based sampling systems in many LMICs, where rapidly expanding settlements often outpace official spatial updates.

The BHDSS baseline findings revealed a significant socio-economic challenge, including widespread insufficient formal education, poverty, high unemployment and poor housing conditions. All these factors contribute to high burden of communicable diseases and this demographic pattern is likely to increase demand for maternal, newborn, child, and adolescent health services, including antenatal care, post-natal care, immunization, nutrition programs, and treatment of common childhood illnesses. It also highlights the need for sustained investment in primary healthcare services to meet current and future population health needs.

The BHDSS WASH and household data highlight considerable environmental health risks. Despite the widespread availability of improved water sources, reliance on solid fuels for indoor cooking remains practically universal, and sanitation primarily depends on pit latrines. This combination signifies considerable exposure to indoor air pollution and potential fecal-oral disease transmission pathways. The use of biomass like woods, grass, and shrubs for cooking exposes households to indoor air pollution, and increase the risk of respiratory illnesses and other serious health outcomes. It also indicates underlying socio-economic vulnerability. Specifically, financial constraints due to unemployment may restrict access to healthcare services; limit health literacy, reduce awareness and utilization of preventive health services, and contribute to delays in seeking appropriate healthcare especially among women and children.

The youthful population structure, socio-economic challenges, and poor environmental conditions imply a population with substantial health needs and vulnerabilities. These findings emphasize the importance of strengthening maternal and child health services, improving health education, and promoting cleaner household energy sources to improve overall population health in the Bauchi HDSS.

The formation of BHDSS in one of Africa’s most populous countries faced numerous challenges and provided insights that may inform future similar initiatives in other areas of Nigeria and Africa. The strong collaboration and partnership at the institutional level with constituted authorities, including the Bauchi State Ministry of Health, the Bauchi State Primary Healthcare Board, and the Local Government Areas of Bauchi and Ganjuwa, provides an avenue for securing governmental legitimacy and operational support. The robust local community engagement, functional longitudinal system covering about 23,476 households with over 151,672 individuals over the period is an indicator of the feasibility of the project. The successful overcoming of mapping enumeration area and boundaries challenges indicates that the HDSS is operationally achievable and sustainable with the right approach.

### Key challenges, lessons learned, and mitigation strategies during implementation

#### Sampling and geographical validity

The sampling and coverage at baseline were informed by the use of enumeration area (EA) data from the 2005 National Population Commission demarcation, as updated EAs were not available. Lacking updated official EA names and codes, some more recent areas/settlements were left out during enumeration and there were instances of boundaries overlapping for some enumeration areas, which led to households being counted twice. However, these challenges were addressed through preliminary fieldwork with all surveillance areas reconstructed into contiguous clusters. Community leaders verified the new EA names, and unique EA codes were made for regions that had not been represented before. To make sure that the data was spatially correct, geo-tagging and geo-referencing were done. This experience demonstrated the importance of obtaining current information or regularly updated EA data to accurately reflect the geographic distribution and organization of the population.

#### Community engagement and trust

At first, many people in the community, especially those in cities, were hesitant to share information because they didn’t trust the initiative or understand what it was trying to do. This challenge affected initial enumeration as many households within the DSA refused to give consent. In response, there were a lot of media engagement, radio and TV broadcast, Health talks during Ante-Natal Care (ANC) visits on weekly basis to amplify the importance of the surveillance system, community meetings, and leaflet distributions to raise awareness. The target audience was mostly women of reproductive age and heads of households in the communities. The community gatekeepers were actively engaged, especially the Bauchi State Council of Traditional Rulers for Health, as well as religious leaders representing the Muslim, Christian, and traditional faiths, which proved instrumental in fostering trust and securing community members acceptance. This top-down engagement was deliberately extended to the grassroots, where community members were directly sensitized and educated about the project’s objectives, with efforts made to encourage their acceptance and enlist their active participation.

Engagement of field workers within the selected communities fostered a sense of ownership, respect for local values and culture, and consequent increased public trust of the project. Furthermore, the project recruited community Liaison Officers tasked with mediating community rejections, resolving emerging challenges, and sustaining ongoing sensitization. The commitment to continuous community engagement, characterized by persistent follow-up, sensitization, and dialogue, served as a foundational strength in addressing misperceptions and reinforcing the value of the HDSS to participating communities. This challenge showed how important it is to engage local stakeholders and locals in their communities.

#### Population mobility and follow-up

Seasonal farming activities caused people to move temporarily in many rural areas, such as Miya, many households were not present during the baseline enumeration and could not be enrolled. This resulted in incomplete household data. The problem showed the importance of having flexible schedules that take into consideration the availability of the selected population. To make sure that all households were covered, the HDSS team set up follow-up visits during the next rounds of surveillance and kept track of all families that were absent. This challenge indicates that the project should put into consideration, the mobility pattern of the affected communities to better match the activity with the seasonal cycles.

#### Digital system and technical capacity

At the commencement of the project, some technical issues with the data collection system were observed. Contextualizing the system to suit the needs of the BHDSS was an issue. Some key issues included frequent system crashes, restricted options on some devices, and problems with getting GPS coordinates. These problems hampered data collection efficiency and affected the timely implementation. The experience showed how important it is for ongoing technical assistance to address device compatibility and software adaptation to local needs. In solving the problem, a capacity building training was organized for the BHDSS Data Manager and Programmer in Kisumu, Kenya. The goal of the training was to support the BHDSS and improve the functionality of the system. This implies that to make future surveillance more effective, the HDSS system development/deployment, maintenance, data quality control, frequent system checks, and built-up as well as technical knowledge are necessary.

### Strengths and limitations of Bauchi HDSS

The Bauchi HDSS is a pioneer HDSS site in Northeast Nigeria and the second in Northern Nigeria. The site has a number of strengths. Firstly, it’s population coverage of over 151,672 individuals across 23,476 households provides a platform for monitoring demographic events in one of the most resource constrained and vulnerable regions with a weak CVR system. The BHDSS will provide locally generated evidence to inform maternal and child health policies, thereby creating a valuable platform for evaluating public health interventions and informing evidence-based decision-making. In addition, data from the BHDSS can be valuable to support research and career development in relevant fields of Demography, Epidemiology, Bio-Statistics and Public Health. It also provides an environment for longitudinal epidemiologic studies. The major limitation of the BHDSS is that some of the communities in the selected wards are not covered. Clusters were formed to select communities based on their proximity to health facilities. Hence, the demographic surveillance area may not be representative of the entire wards.

## Conclusion

The establishment of Bauchi-HDSS is a significant advancement in bridging the disparity in demographic and health data in Northeast Nigeria. The initial census established a foundation for continuous observation of demographic shifts and health implications. It also revealed significant issues, including outdated enumeration data, community mistrust, and technical difficulties. The Bauchi-HDSS has successfully generated high-quality, community-based evidence that addresses the deficiencies of the CRVS system and fosters evidence-based policy, planning, and research, despite initial challenges. This profile establishes a platform for ongoing and future longitudinal studies about health and demographic transformations within this group.

## Supplementary Material

HDSS paper figures.zip

## Data Availability

The data set for this study is available at figshare doi 10.6084/m9.figshare.32043285 and the other data sets from the Bauchi HDSS can be made available on a reasonable request for interested researchers in line with the study protocol and the program office terms and conditions.

## Open scholarship

This article has earned the Center for Open Science badges for Open Data and Open Materials through Open Practices Disclosure. The data and materials are openly accessible at https://10.6084/m9.figshare.32043285 and https://doi.org/10.12688/verixiv.3178.1.

## Preprint

This manuscript has been uploaded to the verixiv platfoarm.

Muhammad Bose I, Mubarak Dabo M, Abdullahi Kobi M et al. The Establishment of Health and Demographic Surveillance System (HDSS) in Bauchi State North East Nigeria; Baseline Findings, Challenges and Lessons Learned [version 1]. VeriXiv 2026, 3:167 (https://doi.org/10.12688/verixiv.3178.1).
